# Supplementary material for: Optimization and Application of CRISPR/Cas9 Genome Editing in a Cosmopolitan Pest, Diamondback Moth
Source: Int J Mol Sci. 2022 Oct 27;23(21):13042. doi: 10.3390/ijms232113042 (PMC9657529; doi:10.3390/ijms232113042)
Supplement: Supplementary file 1 [file ijms-23-13042-s001.zip › ijms-1912474-supplementary.pdf]

Table S1. Primers for amplification of potential promoters of endogenous genes in DBM.

potential promoters	location	Actual length/length in the genome sequence file	Forward primer (5'→3')	Reverse primer (5' →3')
515-1	Upstream of Px02C00515	1131/1131	TTCAAGTAAGAATAACCAAATACATACCCTTCT	TGCCTAGTTCCTATGCGTGAT
515-2	Downstream of Px02C00515	399/3000	GGGTGTGGTGCGGTTTCCTTG	ATTCCCAACGTGGTGATCCGTCAAAAG
303	Upstream of Px05C00303	877/1008	TTCGCACACTTTGTATCACTTCAC	TATAAATAAGAAATAAATGTGAGAGGTCATCAAAT
656-1	Upstream of Px07C00656	676/640	CCAAACAGACTGTAAAAACTGTG	ATTATAATTTTATTTATAGGTACTGATTTTACTGTTATT
656-2	Downstream of Px07C00656	417/1316	TTGGATTGTTCTAATAGCTCGCTAC	TCATTTTAGCCCTAAGATTTTTTACTC
217	Upstream of Px18C00217	1702/1702	ATTTATTTTTTAATTAGGATAATTAATTAGTTTTTAT TCCTG	CTTGATCTGTAACAATGGAATAAAAATGTTAAT
336-1	Upstream of Px18C00336	1600/1600	ATGTTAAATACCGCGTCAGGAAC	ATACAACGTTTGAATCACATTACCAC
336-2	Downstream of Px18C00336	1607/1607	CCACCTCGCAGACGCAGC	CTTCGAGCCGCAACACAC

Table S2. Primers for amplification of the 217 promoter.

Truncated promoter	The truncated length/Total length	Forward primer (5'→3')	Reverse primer (5'→3')
217-1	1462/1702	ATTGGTAAATACCCAGTCTGAGTG	CTTGATCTGTAACAATGGAATAAAAATGTTAAT
217-2	1267/1702	CGGTACCTATCTCTCAAGGCAC	CTTGATCTGTAACAATGGAATAAAAATGTTAAT
217-3	1079/1702	CTGGCTGTACTATGTACTCTGTG	CTTGATCTGTAACAATGGAATAAAAATGTTAAT
217-4	850/1702	AACGCATCGCATAGTAAACTCAC	CTTGATCTGTAACAATGGAATAAAAATGTTAAT
217-5	671/1702	GAGAACTGCGCAGTGCAG	CTTGATCTGTAACAATGGAATAAAAATGTTAAT
217-6	477/1702	CTCTACAATAGGTGTGGCAAGG	CTTGATCTGTAACAATGGAATAAAAATGTTAAT
217-7	263/1702	ATGTTCAGTGTACGTTCTTAGG	CTTGATCTGTAACAATGGAATAAAAATGTTAAT

Table S3. Primers for construction of pGL3 plasmid with endogenous promoters.

Primer	Sequence (5'→3')
pGL3-Basic-reverse amplification-F	TTGGCATTCCGGTACTGTTGGT
pGL3-Basic-reverse amplification-R	ACTTAGATCGCAGATCTCGAG
Homology arm added to endogenous promoter forward primer	ATCTGCGATCTAAGT
Homology arm added to endogenous promoter reverse primer	GTACCGGAATGCCAA

Table S4. Primers for construction of pB-EGFP plasmid with different promoters.

Primer	Sequence (5'→3')
pB-EGFP-reverse amplification-F1	ATGGTGAGCAAGGGCGAGGAG
pB-EGFP-reverse amplification-R2	AGATCTTAATACGACTCACTATAGGGC
Homology arm added to endogenous promoter forward primer	TGAGTCGTATTAAGATCT
Homology arm added to endogenous promoter reverse primer	TCGCCCTTGCTCACCAT

Table S5. Primers for construction of pB-Neo-EGFP plasmid.

Primer	Sequence (5'→3')
Neo-F	ATGATTGAACAAGATGGATTGCACGCAG
Neo-R(HA)	ACCGCATGTTAGAAGACTTCCTCTGCCCTCGAAGAACTC GTCAAGAAGGCGATAGAAG
pB-EGFP-reverse amplification-F2(HA)	CTTCTAACATGCGGTGACGTGGAGGAGAATCCCGGCCC TATGGTGAGCAAGGGCGAGGAGCTGTT
pB-EGFP-reverse amplification-R2	AGATCTTAATACGACTCACTATAGGGC
IE1-F(HA)	TCTTGTTATAGATATCCGATGTCTTTGTGATGCGCGCGA CATTTT
IE1-R(HA)	CATCTTGTTCAATCATCTTGGTTGTTCACGATCTTGTCGC

The underlined part in the table indicates the homology arm added to the primer.

Table S6. Primers for construction of pB-Cas9-Neo plasmid.

Primer	Sequence (5’→3’)
pB-Neo-reverse amplification-F	ACGCGAGTTAATTAAGACCCGGGCTGCAGG
pB-Neo-reverse amplification-F	AGATCTTAATACGACTCACTATAGGGCGAATTGG
Cas9-F(HA)	CCATTGTTACAGATCAAGATGGACAAGAAGTACTCCATTGGGCT
Cas9-R	TCACACCTTCCTCTTCTTCTTGG
217-2-F(HA)	ATAGTGAGTCGTATTAAGATCTCGGTACCTATCTCTCAAGGCAC
217-2-R	CTTGATCTGTAACAATGGAATAAAAATGTTAAT
SV40-F(HA)	AGAAGAAGAGGAAGGTGTGAGATCCACCGGATCTAGATAACTG
SV40-R(HA)	TCTTAATTAACTCGCGTTAAGATACAT
pB-Cas9-detection-F	ATGGACAAGAAGTACTCCATTGGGCTC
pB-Cas9-detection-R1	TCACACCTTCCTCTTCTTCTTGGGGTCAGCCC
pB-Cas9-detection-R2	CCACTACGTGAACCATCACCCTAATC

The underlined part in the table indicates the homology arm added to the primer.

Table S7. Primers for construction of pU6-shRNA (EGFP) plasmid and RT-qPCR.

Primer	Sequence (5’→3’)
Oligo-shEGFP-1	TAGTGTACAACTACAACAGCCACAAGTGTGCTG
Oligo-shEGFP-2	AGGACAGCACACTTGTGGCTGTTGTAGTTGTAC
Oligo-shEGFP-3	TCCTTGTGGCTGTTGTAGTTGTACTTTTT
Oligo-shEGFP-4	AAACAAAAAGTACAACTACAACAGCCACA
EGFP(qPCR)-F	GAACCGCATCGAGCTGAAGG
EGFP(qPCR)-R	CTGCCGTCCTCGATGTTGTG
RPL32(qPCR)-F	CAATCAGGCCAATTTACCGC
RPL32(qPCR)-R	CTGGGTTTACGCCAGTTACG

Table S8. Primers for construction of pU6-gRNA plasmid and amplification assays.

Primer	Sequence (5’→3’)
Oligo-sgRNA(EGFP)-1	TAGTGGCGAGGGCGATGCCACCTA
Oligo-sgRNA(EGFP)-2	AAACTAGGTGGCATCGCCCTCGCC
Oligo-sgRNA1(Ubx)-1	TAGTGGTGGTGGCGAGCAGCAGAA
Oligo-sgRNA1(Ubx)-2	AAACTTCTGCTGCTCGCCACCACC
Oligo-sgRNA2(Ubx)-1	TAGTGGATTCGCCTTACGACGCGT
Oligo-sgRNA2(Ubx)-2	AAACACGCGTCGTAAGGCGAATCC
pU6-detection-R	TCACCAGCGTTTCTGGGTGAG
Ubx-detection-F	CGGGGACCAGTACCGGGGGTTC
Ubx-detection-R	AGAGGAAGAAATCATGGGCTG

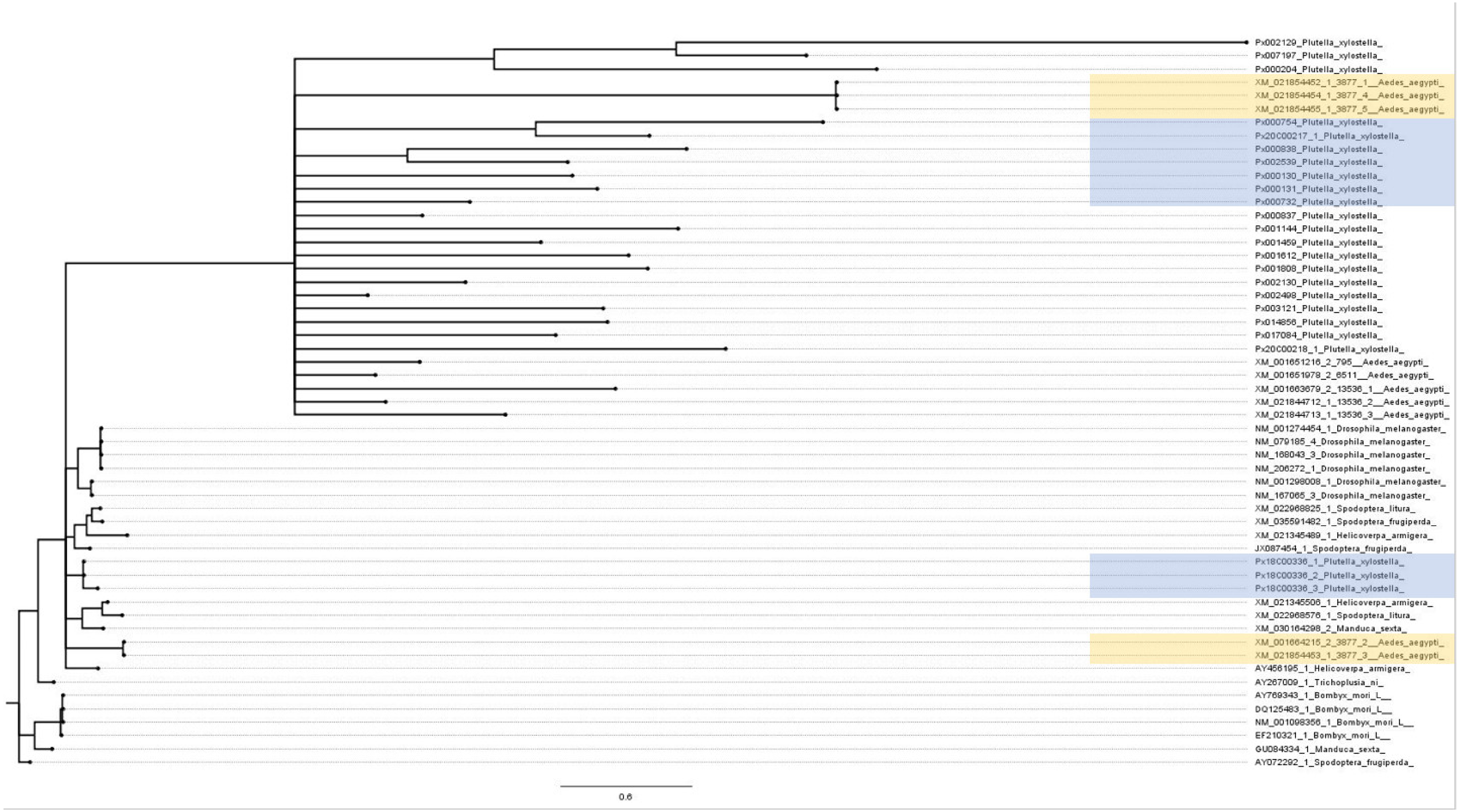

Figure S1. A phylogenetic tree of *polyubiquitin* (*Pub*) genes from different species. The corresponding tree scale is provided in the figure. The region covered in orange is the *Pub* gene with a strong promoter identified in *Aedes aegypti*. The blue area is the selected *Plutella xylostella* endogenous gene with a strong promoter.

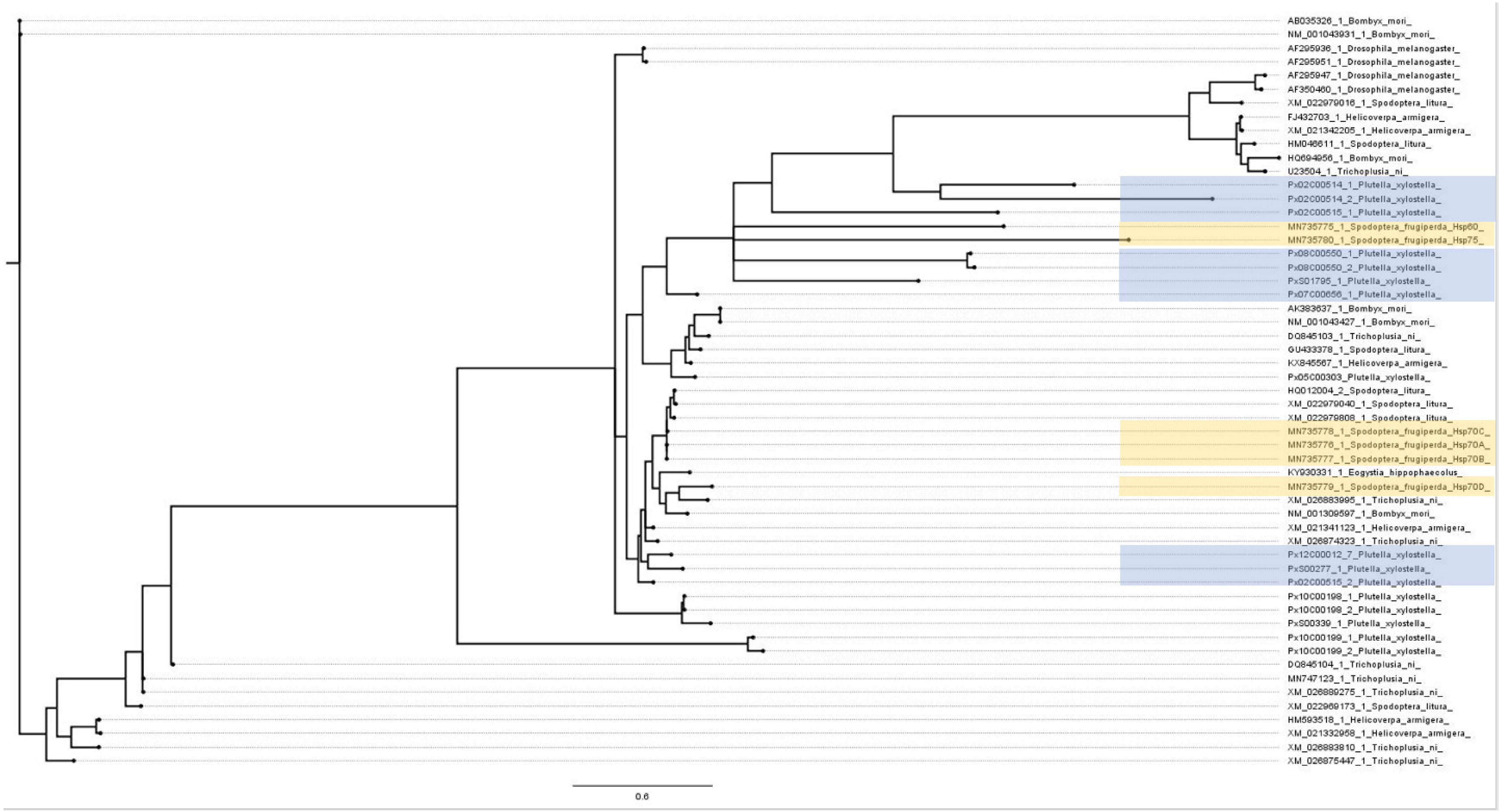

Figure S2. A phylogenetic tree of heat shock protein 70(*Hsp70*) genes from different species. The corresponding tree scale is provided in the figure. The region covered in orange is the *HSP70* gene with a strong promoter identified in *Spodoptera frugiperda*. The blue area is the selected *P. xylostella* endogenous gene with a strong promoter.

-1267 CGGTACCTATCTCTCAAGGCACAGTCTAGTTTAAATAATCAGCTAGACACGACAGATAGATCTTCGCCCCATTTGTATAA
Sp1

-1187 GCCATTTTTATTTAGGAAGGAAGATATAAATTAAGAAAATGTACAATGTTCGTTGGTTCGTGCGAACAGCGATGTTATTA
C/EBPalp TBP

-1107 TTCTGAGATCACGAATTACCAACATTCA**CTGGCTGTACTATGTACTCTGTGATCATGAGTTTGCACTCTTTGCTCTCAAG**
v-Myb C/EBPalp

-1027 **ACATGACGCATGCGTTACTAGAAAAATCAAAATAATCTGCTGTCTCTTTCGTTTGTGCTAAGTGTAAAAGGGAGACGGAT**
C/EBPalp TBP

-947 **AAACAACGGCACCAATCAAAACACAGTATTGATTAAGTTGCAAGTCTATATAAAGCGAGGCAACTATTTCTTGACGCAT**
C/EBPbeta TBP

-867 **TTCAAAGTGAAAAATTA**AACGCATCGCATAGTAAACTCACTGTTGTGTTGAAGATTTTTATTTGTACAAGAACAGACATT
HNF-3

-787 TCCTTGATAAATTATAGTGAATATTGGTAAGTTTCTTTTCCATTCTAACTACTCATAATACACCATACTTAAAATTAGGC
C/EBPalpha

-707 TGAATCTCAAACCAAACTACATTTCTTTCGCTGGCTGAGAACTGCGCAGTGCAGCGCATTTCATTGATTTTCTTTTCCTT
E1

-627 TGTCCGCCATCTTAATCATCTTGCTGGAAAATAAATTGAATCACACCATCGTTTATGCCCTCATCAAGGATTGAATAAAC
C/EBPalpha Sp1

-547 ACAATTTATTGTGGAAATGTTATTGATGATAAACCAACTTAGATTCTCGGATTTTATATAAATTGCAAAACTCTACAATA
TBP

-467 GGTGTGGCAAGGTTAATATAACAGCAATTTACTGCAGAATTTTCTAAAATATTGTAGCTTGCCTAACCTCGTTTTGGTTT
GCBox NF-1

-387 GTGGATCATCTTGGGTTTATTCATATATATAGGTATTACTTACTACCTACGTAGTTACAGCAGTACCTACCTATCAAGTA
GATA-1

-307 ACCCAGAAGAAATATAGGTATAAATAGGTACCTATAACAATATTATGTTCAGTGTACGTTCTTAGGTATTCTAAAAGTAG
CDC5 Evi-1 TBP

-227 GTTTTTAGGTCATTGATTTGAATAGTTCCTTGACCTGTGCAAAACTTCCGTTTGGCTCAGCGCCATTATTATGACGCAGA
GAATbox

-147 CATGTTATGATAGCACAATTCAATAATATTGTGAAATCTTTATCTTTAACACTTATTGAATATAAGTAGGTATGTTTACG
Mat1-Mc TBP

-67 AATAGTGTTATCATCACTTTGTTGATAAAGGGATATTAACATTTTATTCCATTGTTACAGATCAAG***ATG***CAGATCTTTG
TATAbox **+1**

+14 TCAAGACATTGACTGGCAAGACCATCACTCTAGAGGTGGAGCCTGCTGACACCATCGAGAATGTGAAGGCTAAGATTCAG

Figure S3. Characterization of the 217-2 promoter. Potential transcriptional elements in the sequences are predicted by using Genomatix Software Suit and Alibaba 2.0 databases. The bolded sequence part is the transcriptional regulatory region of the core of the 217 promoter, and the ATG marked in red is the translation initiation site of the *Px000217* gene.
